# Bedside shift report: Nurses opinions based on their experiences

**DOI:** 10.1002/nop2.755

**Published:** 2020-12-30

**Authors:** Joseph Jimmerson, Patricia Wright, Patricia A. Cowan, Tammy King‐Jones, Claudia J. Beverly, Geoffrey Curran

**Affiliations:** ^1^ University of Arkansas for Medical Sciences Little Rock AR USA; ^2^ Central Arkansas Veterans Healthcare System Little Rock AR USA

**Keywords:** bedside shift report, hand‐off, nursing report, patient hand‐off, shift report

## Abstract

**Background:**

Nurse bedside shift report (BSR) improves satisfaction, quality and safety. Yet, postimplementation adoption rates remain low in hospitals where BSR has been introduced. Further research is needed to understand what content is most appropriate to discuss during BSR and what facilitators are from the clinical nurses' perspective.

**Aims:**

Identify and describe acute care clinical nurses' and nursing supervisors' experiences and opinions regarding: process of BSR, appropriate content for BSR and barriers and facilitators related to implementation of BSR.

**Design:**

A phenomenological qualitative study was conducted at an acute care 500 bed, not‐for‐profit academic medical centre located in the southern United States.

**Methods:**

Clinical nurses (*N* = 22) and nursing supervisors (*N* = 12) from every inpatient division were recruited and interviewed. The data were analysed for relationships, similarities and differences. Themes were then identified by two independent researchers.

**Results:**

Five themes were identified: (a) time constraints and clinical nurse's workflow must be taken into consideration; (b) a modified approach is necessary; (c) process and specific critical content should be individualized so that it is meaningful for all parties involved; (d) specific critical content that should be discussed outside the patient's room; and (e) specific critical content that should be discussed inside the patient's room.

**Conclusions:**

One way to minimize interruptions is to conduct BSR using a modified approach, where a portion of the hand‐off occurs inside and outside the patient's room. In addition, this study identified the nurses' preferred location where specific critical topics should be discussed.

**Relevance to clinical practice:**

Results from this study should be used to inform the practice BSR so the desired outcomes of patient and family satisfaction, nursing quality and patient safety can be realized. This study should influence future research aimed at identifying strategies for successful implementation and sustained use of BSR. The COREQ checklist was used to write manuscript.

## 1 INTRODUCTION

In 2016, it was estimated that there were 251,000 preventable deaths per year making medical errors the third leading cause of death in the United States (Makary & Daniel, 2016). The exact cost of these medical errors is unknown, but studies estimate they cost the United States economy up to $19.5 billion each year (Milliman, 2010). Finding from a study conducted by the Joint Commission’s Annual Report on Quality and Safety (2007) found that 70% of serious medical errors are a direct result of some type of breakdown in communication from one caregiver to another in what is known as the ‘hand‐off’ process (Joint Commission’s Annual Report on Quality & Safety, 2007). As a result, nurse bedside shift report (BSR) has been recommended by national patient organizations as the gold standard (AHRQ, 2013; Joint Commission, 2015).

Nurse bedside shift report (BSR) has been identified as the gold standard because outcomes reported in the literature indicate it improves patient and family satisfaction, nursing quality and patient safety better than the traditional hand‐off outside the patient's room (Grimshaw et al., 2016). BSR occurs at the patient's bedside where patients and their families can participate in the hand‐off of critical content. Unfortunately, BSR is occurring inconsistently and infrequently across many acute care hospitals that have attempted to implement the practice. Fifty‐one per cent of 653 hospitals recently surveyed acknowledge that significant patient details are frequently not transferred between caregivers during the hand‐off process (Agency for Healthcare Research and Quality (AHRQ), 2014).

## 2 BACKGROUND (LITERATURE)

Nursing hand‐off requires the transfer of critical information from the off‐going nurse to the oncoming nurse. A successful transfer of this information is required to ensure the continuity of patient care and to prevent adverse events and medical errors (AHRQ, 2013). In 2006, the Joint Commission published a national patient safety goal that required organizations to have standardized approaches to hand‐off and encouraged the active participation of patients in the process (Joint Commission, 2015). Following suit, AHRQ published an implementation handbook for BSR which included a checklist of items that should be discussed during the BSR (AHRQ, 2013). Table 1 represents an abbreviated version of AHRQ's checklist.

**TABLE 1:** AHRQ bedside shift report checklist

AHRQ (2013) Bedside Shift Report Checklist	
(1)	Introduce and invite patient and family to participate
(2)	Open electronic medical record in patient's room
(3)	Conduct a verbal report of the following: situation, background, assessment, and recommendation
(4)	Conduct a focused assessment of patient and room (i.e. wounds, incisions, drains, IV sites, IV tubings, catheters)
(5)	Review tasks that need to be done (i.e. laboratories, tests, medication administration, forms)
(6)	Identify if the patient or family have any needs or concerns

In 2017, the Joint Commission issued a sentinel event alert related to inadequate hand‐off communication (Joint Commission, 2017). The alert recommended the critical content that should be communicated during every hand‐off and that hand‐off should occur in a location free from interruptions and include the patient and family as appropriate (Joint Commission, 2017). Table 2 lists the critical content recommended by the Joint Commission.

**TABLE 2:** Critical content to include in hand‐off communications

Critical Content Recommended by Joint Commission (2017)	
(1)	Sender contact information
(2)	Illness assessment, including severity
(3)	Patient summary, including events leading up to illness or admission, hospital course, ongoing assessment, and plan of care
(4)	To‐do action list
(5)	Contingency plans
(6)	Allergy list
(7)	Code status
(8)	Medication list
(9)	Dated laboratory tests
(10)	Dated vital signs

Although studies in the literature have begun to investigate nurses' experience and opinions regarding of BSR, the most feasible process to conduct BSR and the specific critical content that should be included in BSR has yet to be fully defined. This study is the first to define specific critical content as unambiguous items that should be discussed or performed during BSR (i.e. an assessment of the patient's intravenous catheter is performed, a decision is made that the patient needs pain medication and the patient‐controlled analgesia settings are checked to ensure they match the physicians order). Staggers and Jennings (2009) observed hand‐off in a variety of different formats, recorded audio tapes, face‐to‐face and BSR and did describe a broad overview of the content they observed. High level overview of content is defined as overall themes of information that are discussed or performed during BSR (i.e. an assessment is performed, decisions are made, information is clarified, errors are intercepted and care is prioritized). Staggers and Jennings (2009) reported the following (a) 33% was clarifying details (i.e. exchanging questions and answers between the oncoming and off‐going nurse), (b) 30% was factual information (i.e. patient' name, bed number, age, weight, laboratory tests, physician orders, locations of intravenous lines, tubes and drain locations and times medications and treatments were completed and/or due.), (c) 25% was nursing actions, knowledge, judgments and instincts combined with decisions; and (d) 13% was teamwork, relationship building and smoothing the transition of care (i.e. humour and laughter).

The aim of this study was to identify and describe acute care clinical nurses' and nursing supervisors' experiences and opinions regarding: process of BSR; appropriate content for BSR; and barriers and facilitators related to implementation of BSR. This article compares and contrasts the hand‐off expectations set by Joint Commission (2017), AHRQ (2013) and the findings of this study. Unlike current studies in the literature, this study explored BSR in a high acuity academic medical centre environment. This study is also unique from other BSR studies because an implementation science framework, iPARIHS, was used to design the interview guide and analyse the findings. According to its creators, Kitson and Harvey (2016), the iPARIHS framework was designed to explain and predict the success of implementation of evidence into practice. The core constructs of the iPARIHS framework are innovation, recipients, context and facilitation (Kitson & Harvey, 2016). The interpretation of the iPARIHS constructs as they relate to this study were the innovation was BSR, the recipients were nursing supervisors and clinical nurses, the outer context was hospital administration, the inner context was the nursing culture and facilitation was the strategies used to implement BSR at the study site. The iPARIHS framework was chosen as the sensitizing framework for this study because it accurately represents the complexities of implementing BSR, helped to explain why BSR has not been successfully implemented and was useful to determine the key elements necessary to implement BSR successfully in the future. An excerpt from the in‐depth interview guide which includes only the main questions and which iPARIHS concept(s), was used to develop them can be found in Table 3.

**TABLE 3:** Excerpt: In‐depth interview guide

Excerpt: In‐depth Interview Guide		
Main Questions (1st question is grand tour question)	Specific Aim(s)	iPARIHS Concept(s)
(1) Tell me about shift change report in your unit	1, 2, 3	All
(2) Tell me about your experience with shift change report at the bedside at this hospital	1, 2, 3	All
(3) Tell me about your experience with shift change report at the bedside at other hospitals you've worked	1, 2, 3	All
(4) What topics do you think should be discussed at the bedside?	1,2	
(5) Why do you think hospital administration wants change of shift report to be at the bedside?	3	Innovation, outer context
(6) What value do you place on conducting change of shift report at the bedside?	3	Recipients
(7) How receptive is your unit to report at the bedside?	3	Recipients
(8) Describe how change of shift report at the bedside was implemented on your unit?	3	Facilitation, inner context, innovation
(9) What worked well versus what didn't? Why?	3	Facilitation
(10) What would help nurses implement bedside reporting (facilitators)?	3	Facilitation

## 3 METHODS (DESIGN, DATA COLLECTION AND ANALYSIS)

### 3.1 Research design

To fulfil the purpose and specific aims of this research study, the investigators needed to understand the intricacies of BSR from both the frontline nurses and their supervisors who have tried to implement it. Therefore, a qualitative methodology, phenomenology, was used to explore the essence of the phenomenon, BSR.

### 3.2 Setting

This study was conducted at an acute care 500 bed, not‐for‐profit academic medical centre located in the southern United States. The medical centre employees approximately 1,000 acute care adult clinical nurses. The racial and gender distribution for those clinical nurses is as follows: 16.05% Black African American, 1.74% Hispanic, 0.69% American Indian/Alaskan, 3.23% Asian/Pacific Islander/Hawaiian, 77.14% White, 1.15% other/unknown, 13.45% male and 86.55% female. There are 15 acute care adult inpatient units at the study site, and each one is managed by a nursing supervisor. Those units are divided into the following divisions: medical surgical, oncology, maternal infant and critical care. The racial and gender distribution for study site nursing supervisors (unit managers) was as follows: 27% Black African American Female, 60% White Female and 13% White Male. The investigators are employed at the research site and has access to the study population. Prior to recruiting, permission was obtained from the medical centre's Chief Nursing Officer, Associate Chief Nursing Officer and the Institutional Review Board (IRB). BSR implementation was attempted at the study site approximately 7 years prior to this study being conducted.

### 3.3 Sample/Participants

Phenomenological research studies generally use purposeful sampling methods to ensure that participants are information rich (Butcher, 2019). This study included clinical nurses and their supervisors who had firsthand experience with BSR to see if supervisors had a different perspective than clinical nurses.

#### 3.3.1 Clinical nurses

A purposive sample of 22 clinical nurses was recruited from a variety of acute care adult inpatient units at the academic medical centre via a recruitment email sent by the principal investigator. For the purpose of this study, a clinical nurse is defined as a registered nurse who spends 50% or more of their work time delivering direct patient care. This sample size was based on recommendations for phenomenological studies and was consistent with other like qualitative studies (Creswell & Poth, 2018; Grimshaw et al., 2016). Although the anticipated maximum sample size was 30, saturation was achieved at 17 participants. However, recruitment continued until a minimum of one clinical nurse from each of the 15 inpatient units was recruited and interviewed. All clinical nurses who expressed interest in the study and met eligibility criteria was interviewed.

Eligibility criteria for participation of the clinical nurses included (a) age 18 years or older; (b) registered nurse who spent 50% or more of their work time delivering direct patient care; (c) were currently working on an acute adult care inpatient unit at the medical centre; and (d) had experienced/attempted BSR. Nurses were not eligible to patriciate in this study if they had not completed employee orientation or were in a supervisory position. These criteria were selected to ensure the participants had an opportunity to fully experience the phenomenon of BSR as a clinical nurse.

#### 3.3.2 Nursing supervisors

A purposive sample of 12 nursing supervisors was recruited from the same 15 acute care adult units at the medical centre. Nursing supervisors were recruited via a recruitment email sent by the principal investigator. This sample size was consistent with recommended sample sizes for phenomenological studies and aimed to recruit a minimum of 50% of the study population (Creswell & Poth, 2018). Although the anticipated minimum sample size was 8, recruitment and data collection continue until saturation was achieved at 12 participants. A minimum of one nursing supervisor from every inpatient division at the study site was recruited and interviewed.

Eligibility criteria for participation of the nursing supervisors included (a) 18 years of age or older, (b) currently supervising one of the medical centre's acute care adult inpatients units and (c) had experienced/attempted implementing BSR. Nursing supervisors were not eligible to participate in this study if they had been in a supervisory position at the medical centre for less than 1 year. Criteria were established to ensure the participants had an opportunity to fully experience the phenomenon of implementing BSR in a supervisory capacity. Clinical nurses and nursing supervisors were included as participants to capture the opinions of both groups regarding barriers and facilitators to BSR.

### 3.4 Participant recruitment

The lead investigator was employed at the research site and had access to the study population. Prior to recruiting, permission was obtained from the medical centre's Chief Nursing Officer, Associate Chief Nursing Officer and the Institutional Review Board (IRB). The principal investigator sent out a recruitment email to all inpatient acute care nurses employed at the medical centre and posted flyers on all acute care inpatient nursing units. However, the investigators were unable to recruit enough participants via this method, so the principal investigator and the Associate Chief Nursing Officer informed nurses at various nursing meetings of the opportunity to participate. They explained that participation in the research study was voluntary and consisted of a face‐to‐face interview and completion of a demographic data sheet. The principal investigator answered and explained all questions potential participants had. Nurses interested in participating were asked to contact the principal investigator via email. The investigators were not made aware of any potential participants via any other routes.

### 3.5 Obtaining consent

Prior to the interview, a waiver of written consent was requested and obtained from the IRB. An information sheet was provided to the participant to ensure the participant understood the purpose of the study, what the findings were to be used for and that they had the right to withdraw from the study at any time without consequence. Consent was considered obtained when: the nurse had reviewed the information sheet and verbally agreed to participate. The IRB did not require written consent because there was no more than minimal risk to participants.

### 3.6 Data collection

Participants completed a demographic questionnaire prior to each interview. The questionnaire captured personal information, registered nurse experience, BSR experience, registered nurse education and work information. In‐depth interviews were conducted using an interview guide. The interview guides include broad open‐ended questions that are strategically ordered and connected to ensure that valuable information was obtained throughout the interviews (Rubin & Rubin, 2012). The selection of these questions was influenced by the findings of the prior qualitative studies already mentioned, two pilot studies conducted by this principal investigator (Jimmerson, 2017, 2018) and the concepts of the iPARIHS framework. The questions were also chosen based off their ability to fulfil the aims of the study. In addition to the main questions listed on the interview guides, additional probing, clarifying and redirecting questions were asked as necessary to ensure rich data information is obtained (Creswell & Poth, 2018).

### 3.7 Conducting the interview

The principal investigator contacted the identified participants to determine a mutually agreed time and place to conduct an in‐depth face‐to‐face interview. Interviews occurred on the hospital campus. The interviews occurred in a quiet room that was located in a different building than the inpatient unit to protect the confidentiality of the participant. All interviews occurred at times that participants were not being paid by their employer (i.e. before or after scheduled shift). The length of the interviews lasted approximately 30–60 min and were audio recorded on a password encrypted device. Only the principal investigator and the participant where in the interview. In addition to the using the interview guide, the principal investigator also took field notes immediately after each interview. The field notes were used to capture participants' non‐verbal ques not captured via audio recording. The field notes were used to assist the investigators with analysis and interpretation of data. Probing, clarifying and redirecting questions were asked during the interview to ensure rich data information was obtained (Creswell & Poth, 2018). At the close of each interview, the principal investigator asked for participant's permission to set up a secondary phone interview in the event that new themes emerge, or additional clarification was needed.

### 3.8 Ensuring dependability, confirmability and credibility

This study was completed in the principal investigator's place of employment where he is in a leadership role. Conducting qualitative research in one's place of employment can produce instant rapport with participants, insights that only an insider may be privy to and better translation of research findings into practice (Josselson, 2007; McConnell‐Henry et al., 2010; McDermid et al., 2014; Moore, 2012; White, 2012). However, challenges associated with conducting research in one's place of employment include: (a) peers and colleagues may feel coerced into participating, (b) the investigator may lose objectivity and (c) participants may not be completely truthful in their responses (Moore, 2012; McConnell‐Henry et al., 2010). However, there are strategies that these investigators leveraged to ensure these risks were mitigated.

The investigators used sampling approaches that mitigated possible coercion, practised reflexivity to ensure objectivity and built rapport with participants to garner truthful responses. Participation was completely voluntary and non‐coercive. They understood their own philosophical beliefs, did not project them into the study and allowed themselves to be challenged by what participants said throughout the course of the research (Moore, 2012). They took a step back at various stages of the research and practiced reflexivity to ensure they was not projecting bias in the study (Moore, 2012). Finally, the investigators made the purpose of the research well known to participants, debunked any rumours of hidden agendas, established clear distinctions between the investigators' role versus their peer/colleague roles and excluded significant information that may de‐identify participants. This ensured that participants felt more comfortable expressing their true experience and opinions regarding BSR (McConnell‐Henry et al., 2010).

To further ensure dependability, confirmability and credibility, the recordings were transcribed verbatim. After the recordings were transcribed verbatim, the principal investigator read the interviews to gain familiarity and to check for accuracy. Once accuracy was verified, two independent investigators classified the data into codes and themes. The codes were then used to determine the emerging themes.

### 3.9 Trustworthiness/Validation

According to Creswell and Poth (2018), qualitative investigators should engage in at least two validation methods to ensure that their study generates accurate and valuable information. The following strategies were used to ensure this study produced valid results: (a) Prolonged engagement and persistent observation. (b) Member checking is the most critical method used to establish credibility (Creswell & Poth, 2018). Throughout the process and at the conclusion of each interview, the principal investigator summarized and used probing questions to clarify important issues to ensure accurate accounts were recorded. Transcripts were not returned to participants. Secondary phone interviews were deemed to be unnecessary. Rich descriptions**—**Specific quotations from interviewees were included in this article. This allows the reader to make their own decisions regarding transferability and accuracy of the conclusions (Creswell & Poth, 2018). The consolidated criteria for reporting qualitative research (COREQ) was used to ensure accurate and complete reporting of this study occurred (Tong et al., 2007). Please see the completed checklist: ‘Consolidated criteria for reporting qualitative research’ (Appendix S1).

### 3.10 Data analysis

The demographic questionnaires were analysed using descriptive statistics. For example, measures of central tendency and variability were calculated for participant age, years of nursing experience and BSR experience. Transcribed interviews were entered into MAXQDA 2020 to facilitate sorting and coding the data. The principal investigator and one other investigator read the first three interviews from each participant sample independently. They identified the main topics of the interviews from each sample and checked for agreement when coding. Together, they established a codebook that included main categories with definitions. This codebook was used to code all remaining interviews by the principal investigator. Once all interviews had been coded, the investigators analysed the data for relationships, similarities and differences (Giorgi, 1985). The emerging themes were identified and those pertinent to the aims of this article were reported in the results section.

## 4 RESULTS

Clinical nurse recruitment stopped after saturation was achieved at *N* = 22 participants. A minimum of one clinical nurse from every inpatient unit was recruited and interviewed. Nursing supervisor recruitment and data collection continued until saturation was achieved at 12 participants. A minimum of one nursing supervisor from every inpatient division was recruited and interviewed. A complete analysis of the demographic data can be found in Tables 4 and 5. All participants expressing interest in the study whom met inclusion/exclusion criteria were interviewed. No participants dropped out of the study.

**TABLE 4:** Demographic data analysis clinical nurses

Population		Age	Gender	Ethnicity	RN Experience	RN Tenure at Site	Highest Degree	Charge Nurse	Preceptor
Participant demographics	Clinical Nurses (*N* = 22)	Mean	86% Female	77% White	Mean	Mean	14% Masters	60% yes	82% yes
		(47 years)		10% African American	(14 years)	(11 years)	50% Bachelors		
		Median	14% Male	10% Hispanic	Median	Median	27% Associates	40% no	18% no
		(49 years)		3% Asian	(10.5 years)	(8.5 years)	9% Diploma		
Study site demographics	Clinical Nurses (*n* > 1,000)	Unknown	87% Female	77% White	Unknown	Unknown	Unknown	Unknown	Unknown
				16% African American					
				2% Hispanic					
			13% Male	3% Asian					
				<1% American Indian					

**TABLE 5:** Demographic data analysis nursing supervisors

Population		Age	Gender	Ethnicity	RN experience	RN tenure at site	Highest degree	Tenure as nursing supervisor
Participant demographics	Nursing Supervisors (*N* = 12)	Mean	83% Female	75% White	Mean	Mean	67% Masters	Mean
		(41 years)			(15 years)	(12 years)		(5 years)
		Median	17% Male	25% African American	Median	Median	33% Bachelors	Median
		(38 years)			(14.5 years)	(13.5 years)		(4 years)
Study site demographics	Nursing Supervisors (*N* = 15)	Unknown	87% Female	73% White	Unknown	Unknown	Unknown	Unknown
			13% Male	27% African American				

Five themes, pertinent to the purpose of this article, were identified from the data collected, which included (a) time constraints and clinical nurse's workflow must be taken into consideration; (b) a modified approach is necessary; (c) process and specific critical content should be individualized so that it is meaningful for all parties involved; (d) specific critical content that should be discussed outside the patient's room; and (e) specific critical content that should be discussed inside the patient's room. Subthemes were identified for each theme.

### 4.1 Theme 1: Time constraints and clinical nurse's workflow must be taken into consideration

According to participants, approximately 30 min is allotted for the off‐going clinical nurses to hand‐off their patients to the oncoming shift. Most clinical nurses reported being assigned five or six patients, which if divided equally equates to approximately 5 min per hand‐off. However, the number of patients assigned varied for some units (i.e. intensive care units reported only be assigned one or two patients depending on severity of illness). Clinical nurses and nursing supervisors reported that completing the entire hand‐off in the patient's room took more time than traditional hand‐off outside the patient's room for a variety of reasons.

#### 4.1.1 Subtheme 1: You have to think Harder about what you are going to Say

We like to be able to speak a little more freely than we can in front of patients. Jargon too, we can abbreviate things more. (CN 5)

I think that the nurses have a lot of information to share in short period of time. And I think quite frankly that they don't want to have to think about what they're saying in front of a patient, they just want to say it. (NS 1)

#### 4.1.2 Subtheme 2: Interruptions take Precious Time and could lead to Important Things being missed

You're trying to think ahead and get through this report and now the patient needs to be cleaned up and they've had a bowel movement, or they didn't empty the Foley and so you're like having to do it real quick and the epidural is dry and so you request another epidural bag from pharmacy and so that that takes time. And it interrupts your flow. (CN 4)

If there's a very involved family it can totally throw off the flow of information and honestly then sometimes that might make it worse, something might get missed because you're not able to go through your head‐to‐toe. (CN 14)

### 4.2 Theme 2: A modified approach is necessary

All 34 participants, without being prompted, reported that a modified approach, where a portion of the hand‐off occurs inside and outside the patient's room, was necessary to complete a successful transfer of critical information from one shift to the next. In addition, participants reported that a modified approach was better that a full hand‐off at the bedside for a variety of reasons.

#### 4.2.1 Subtheme 1: Modified Approach is Better/more Feasible than a Full Hand‐off at the Patient's Bedside

I think the report you get suffers. When you do a complete bedside. Because you don't get the part you don't want the patient to hear. (CN 10)

We focus on what we call shift report essentials. In reality, it's really looking more towards introductions safety checks equipment checks and also the day. They don't really go through like a whole system based report in the patient's presence. I know that we weren't supposed to individualize it that much to our areas but it reality that's how we're successful in our areas. (NS 1)

#### 4.2.2 Subtheme 2: Rehearsal outside the patient's room first, makes for a better hand‐off

I'm all for going in, but I kind of like a little picture before I go in, so I know what I'm supposed to look for. (CN 12)

It should start outside of the patient's room covering the general information history what's occurred, then kind of go through a head‐to‐toe assessment of what's going on. Then you go into the room and you can finish the bedside report at the bed, looking at all of the things that you might have noted. (NS 8)

#### 4.2.3 Subtheme 3: Some topics are not appropriate to discuss in the patient's presence

Oh, you know, has been worrying me a little bit, the blood pressure and you know the patient probably knows about it but then we can talk just a little more directly to each other and then softening it when we talk about the patient. Watch him, you know. (CN 5)

All those extra things, I don't feel like it needs to happen in there. Because then you can open yourself up to saying something that's not right and nurses we're human, you know. (NS 11)

### 4.3 Theme 3: Process and specific critical content should be individualized to ensure it is helpful and meaningful for all parties involved

As aforementioned, nurses reported they have a limited amount of time to conduct hand‐off. According to participants, this time is precious and should not be wasted by discussing content that is not applicable/important to that specific patient. Participants overwhelmingly reported that the critical content discussed during hand‐off should be individualized to ensure it is meaningful for all parties involved.

#### 4.3.1 Subtheme 1: Hand‐off should be individualized to why patient in hospital and what we can do to help

Some of our patients have really long complex medical histories, we really just need to touch base on why they're here and what we can do to help them. (NS 4)

So I think what's discussed is largely dependent on which unit you're on. (NS 5)

#### 4.3.2 Subtheme 2: Waking patients up to participate in BSR may not always be in their best interest

You don't want to wake them up to do it, because if you wake them up then they're going to need more pain medicine or need or nausea medicine. (CN 9)

If they say don't wake me up then we have to honor that. (NS10)

#### 4.3.3 Subtheme 3: What's discussed should depend on severity of illness and oncoming nurses’ prior knowledge of patient

Some of them it's quicker if they don't have a lot going on, some of them, you know, we have a patient with chest tubes and lines it takes longer to get everything but if you've got a walkie talkie, it's like two minutes and you walk in the same room and walk out. (CN 8)

Depends on the patient, how, what all has occurred and how sick they are. And if it's a new nurse or not if it's someone who's, took care of the patient the day before you know there's a lot. You don't have to recover all the history. (NS 8)

### 4.4 Theme 4: Specific critical content that should be discussed inside the patient's room

Participants were asked the following question: ‘What topics do you think should be discussed at the bedside?’ The most common responses to this question from nursing supervisors, clinical nurses and the combined group are summarized in Table 6.

**TABLE 6:** Specific critical content that should be discussed inside the patient's room

Inside the patient's room‐specific critical content	Nursing supervisors % (*N*)	Clinical nurses % (*N*)	Combined % (*N*)
Introductions/communication boards	42% (*N* = 5)	59% (*N* = 13)	53% (*N* = 18)
Diagnosis‐why in hospital (i.e. surgical procedure)	58% (*N* = 7)	32% (*N* = 7)	41% (*N* = 14)
Plan of care/goals for day	67% (*N* = 8)	68% (*N* = 15)	68% (*N* = 23)
Pain/nausea (symptom control‐including medication schedule)	42% (*N* = 5)	45% (*N* = 10)	44% (*N* = 15)
Medications infusing (i.e. TPN, PCA, Chemo, fluid, heparin, magnesium, vasopressor, epidural)	83% (*N* = 10)	77% (*N* = 17)	79% (*N* = 27)
Skin issues/wounds/incisions/dressings/swelling	58% (*N* = 7)	64% (*N* = 14)	62% (*N* = 21)
Vitals‐pulse/NV/SaO2 (specific dx/extremity)	17% (*N* = 2)	41% (*N* = 9)	32% (*N* = 11)
Lines/drains/airways (vascular access, chest tube, biliary, NG, OG, ETT‐vent, tube feeds, oxygen, etc.)	83% (*N* = 10)	95% (*N* = 21)	91% (*N* = 31)
Focused assessment specific to why they are here (i.e. breathing, neurovascular status, weight)	42% (*N* = 5)	55% (*N* = 12)	50% (*N* = 17)
What happened previous shift (bolus, etc.)	0% (*N* = 0)	14% (*N* = 3)	9% (*N* = 3)
Environment (clean, supplies, call light within reach)	0% (*N* = 0)	32% (*N* = 7)	21% (*N* = 7)
Education to prevent harm (i.e. falls, safe sleep)	50% (*N* = 6)	32% (*N* = 7)	35% (*N* = 12)
Armband, allergies	33% (*N* = 4)	5% (*N* = 1)	15% (*N* = 5)
Discharge plan	33% (*N* = 4)	0% (*N* = 0)	12% (*N* = 4)
Questions/concerns	50% (*N* = 6)	18% (*N* = 4)	29% (*N* = 10)

### 4.5 Theme 5: Specific critical content that should be discussed outside the patient's room

Participants were asked the following question: ‘What topics do you think should not be discussed at the bedside?’ The most common responses to this question by nursing supervisors, clinical nurses and the combined group are summarized in Table 7.

**TABLE 7:** Specific critical content that should be discussed outside the patient's room

Outside the patient's room‐specific critical content	Nursing supervisors % (*N*)	Clinical nurses % (*N*)	Combined % (*N*)
History (i.e. abortion, drug/alcohol abuse, sexually transmitted disease)	67% (*N* = 8)	64% (*N* = 14)	65% (*N* = 22)
Diagnosis‐why in hospital (i.e. surgical procedure, poor prognosis, terminal illness, drug/alcohol withdrawal, suicidal/homicidal ideation, dementia, delirium, sexually transmitted disease)	75% (*N* = 9)	68% (*N* = 15)	71% (*N* = 24)
Review of systems/head‐to‐toe assessment	33% (*N* = 4)	45% (*N* = 10)	41% (*N* = 14)
Things not discussed by provider yet (i.e. imaging results, laboratories, treatment plans, diagnosis, prognosis)	33% (*N* = 4)	50% (*N* = 11)	44% (*N* = 15)
End‐of‐life/DNR status/power of attorney	17% (*N* = 2)	5% (*N* = 1)	9% (*N* = 3)
To‐do action list (i.e. laboratories, ADLs, drugs)	17% (*N* = 2)	0% (*N* = 0)	6% (*N* = 2)
Prisoner status	8% (*N* = 1)	0% (*N* = 0)	3% (*N* = 1)
Family drama (i.e. protective orders)	8% (*N* = 1)	36% (*N* = 8)	26% (*N* = 9)
Medications not typically given on the unit	8% (*N* = 1)	0% (*N* = 0)	3% (*N* = 1)
Items requested to not be discussed by patient	0% (*N* = 0)	23% (*N* = 5)	15% (*N* = 5)
Psychosocial issues (i.e. traumatic event leading to hospitalization)	42% (*N* = 5)	23% (*N* = 5)	29% (*N* = 10)
Unvalidated concerns (i.e. nurse's intuition)	0% (*N* = 0)	5% (*N* = 1)	3% (*N* = 1)

## 5 DISCUSSION

The iPARIHS framework facilitated the identification and description of why organizations have struggled to implement BSR successfully. Furthermore, it helped to describe acute care nurses’ experience and opinions regarding the most feasible process to conduct BSR and the specific critical content that should be discussed during BSR. Findings from this study are congruent with recommendations from the Joint Commission and AHRQ that a successful transfer of critical content from the off‐going nurse to the oncoming nurse should occur in a timely fashion. In their BSR implementation handbook, AHRQ (2013) acknowledges that hand‐off should not take longer than 5 min which was consistent with the amount of time provided to clinical nurses at this academic medical centre to conduct hand‐off. This is also consistent with the findings from an observational study that reported the average nursing hand‐off took 4.4 min (Staggers & Jennings, 2009). The Joint Commission (2017), in their sentinel event alert regarding inadequate hand‐off communication, stated that everything needed to safely care for the patient should be communicated in a timely fashion so care is not delayed. The Joint Commission (2017) went on to say that hand‐off should occur in locations free from non‐emergent interruptions to ensure that important information is not lost between the sender and receiver. This study confirms the Joint Commissions position that interruptions take precious time and can lead to important information not being transferred from one shift to the next. AHRQ (2013) did not mention the word interruption in their BSR implementation handbook. However, several qualitative studies identified interruptions as a common occurrence that has detrimental effects on implementing BSR successfully (Grimshaw et al., 2016; Spooner et al., 2015; Staggers & Jennings, 2009; Tobiano et al., 2017). Participants of this study reported that one way to minimize interruptions is to conduct BSR using a modified approach, where a portion of hand‐off occurs inside and outside the patient's room. This finding is further validated by a study conducted by Grimshaw et al. (2016). Clinical nurses in their study reported that a modified approach should be used to decrease the time BSR takes and ensure all important, information, sometimes sensitive and difficult to discuss in front of the patient, is delivered. According to the iPARIHS framework, successful implementation is most likely to occur when recipients are allowed to blend practice‐based knowledge with the innovation (Kitson & Harvey, 2016). Therefore, a modified approach to BSR should be considered.

This study supports the Joint Commission's and AHRQ's position that the patient and family needs be involved in hand‐off communications. However, the extent of patient and family involvement is not well defined by either the Joint Commission or AHRQ. The Joint Commission stated that patients and families should be included in hand‐off as appropriate (Joint Commission, 2017). AHRQ (2013) positions that the hand‐off of critical content should occur at the patient's bedside but acknowledged that sometimes sensitive information, information not yet shared by the doctor or a sensitive diagnosis such as HIV, should be shared prior to entering the room. The findings of this study support the recommendation from AHRQ that some topics are not appropriate to discuss in the patient's presence. This finding is further validated by a qualitative study that found nurses were not able to say everything they wanted to at the bedside for fear of saying something that may upset the patient (Grimshaw et al., 2016). However, this study differed from AHRQ because it found that a modified approach was better, more feasible and made for a better hand‐off than a hand‐off of all critical content at the patient's bedside. Another study similarly reported that participants in their study felt a modified approach was necessary to ensure that nurses could say everything they needed to say in a timely fashion (Grimshaw et al., 2016). An observational study found the average number of interruptions in the intensive care setting was two interruptions per hand‐off with a range of 0–7 (Spooner et al., 2015). Another study did not quantify the interruptions they observed but stated that interruptions were common in face‐to‐face hand‐off (Staggers & Jennings, 2009). In addition, other qualitative studies found that interruptions by patients and families disrupted the flow of information and was perceived as a barrier by participants (Johnson & Cowin, 2012; Tobiano et al., 2017). One study found that nurses can control hand‐off better if done outside of the patient's room, thus leading to less interruptions (McMurray et al., 2010). Other studies purport that nurses perceived they could not say all of the things they wanted to in hand‐off and were forced to censor information in a way that sometimes led to miscommunication of important information (Bruton et al., 2016; Grimshaw et al., 2016; Johnson & Cowin, 2012; Kerr et al., 2014; Khuan & Juni, 2017; McMurray et al., 2010; Tobiano et al., 2017). As aforementioned, the iPARIHS framework purports that recipients should be allowed to blend practice‐based knowledge with the innovation, to increase the odds of successful implementation (Kitson & Harvey, 2016). Therefore, a modified approach to BSR should be considered to help minimize distractions and ensure communication of critical content in a timely manner.

Results support that critical content that should be communicated during the hand‐off process should be standardized (AHRQ, 2013; Joint Commission, 2017). Neither the Joint Commission nor AHRQ mentioned whether the critical content discussed during hand‐off should be individualized to the patient. However, the critical content that should be communicated in hand‐off according to the Joint Commission is broad and leaves much room for interpretation. AHRQ states their recommended BSR checklist can be individualized and they encourage hospitals to adapt the tool to meet their needs. This study supports creating a standardized checklist of critical content to support a highly reliable process for conducting hand‐off communication. However, it also acknowledges the need to individualize the critical content discussed to ensure it is helpful and meaningful for the nurses, patient and family. This finding is supported by other qualitative studies in the literature. One study found that hand‐off should be flexible to produce good communication and should be based on the complexity of the patient (Johnson & Cowin, 2012). Another study found that although everyone should understand their role in the process of hand‐off, it does not need to be uniform in all clinical areas (Bruton et al., 2016). Another study purported that a consistent and tailored structure was needed for an effective hand‐off process but that it should be unique to the unit (Staggers & Jennings, 2009). Findings from this study are consistent with AHRQ's that patients should be excluded in the discussion portion off hand‐off if desired; items requiring visualization should still occur in the patient's room. This finding is further validated by a qualitative study that purported the following: the decision to conduct BSR should be left up to the patient (Khuan & Juni, 2017).

One finding from this study was that the participant's perspective regarding critical content that should be included in the hand‐off process was congruent between the participants of this study, the Joint Commission (2017) and AHRQ (2013). While the Joint Commission and AHRQ provided broad descriptions of the critical content that should be included, this study identifies the specific critical content that should be included in hand‐off. Although most critical content identified by nursing supervisor and clinical nurse participants was similar, there were a couple of differences. For example, 32% of clinical nurse participants (*N* = 7) reported that assessing the environment was important while nursing supervisors failed to include it as a critical component of BSR. In addition, 33% of nursing supervisors (*N* = 4) reported that discharge plans were important, but it was not mentioned by clinical nurse participants.

The biggest limitation of this study was that it occurred in a single study site. As with any qualitative study, the generalizability of the findings is limited. Since this study focused on inpatient acute care settings, the results may not be transferable to other hospital settings (i.e. emergency department). The data were obtained from nurses' self‐report of their experiences with implementation of BSR. Therefore, there is a potential for recall bias. Another significant limitation of this study is that patients and their family members were not interviewed as part of this study. Research suggests that that their engagement in this process in important to its success (AHRQ, 2013). However, BSR is occurring inconsistently and infrequently making it difficult to explore patients and families' experiences regarding BSR (Agency for Healthcare Research and Quality (AHRQ), 2014). One study concluded that there is confusion among nursing personnel regarding the meaning and purpose of BSR therefore making it difficult to explore patients and families' experiences (Bruton et al., 2016). Once patients and families have had an opportunity to experience BSR as intended, future research should be conducted with patients and families to ensure BSR is meaningful for all parties involved in the process. For the purposes of this study, the following probing question was asked of participants: How involved are the patient and family in shift change report? Future research will incorporate patients and families as participants to ensure BSR is meaningful for all parties involved.

## 6 CONCLUSION AND RELEVANCE TO CLINICAL PRACTICE

The iPARIHS framework was useful to identify the most feasible process to conduct BSR and the specific content that should be discussed during BSR. This article compared and contrasted the hand‐off expectations set forth by the Joint Commission, AHRQ and the findings of this study. In this study, the Joint Commission and AHRQ all agree that a successful transfer of critical information from the off‐going nurse to the oncoming nurse should occur in a timely fashion, the patient and family should be involved in hand‐off communications as appropriate and the critical content that should be communicated during the hand‐off process should be standardized and individualized to the institution.

This study supports the Joint Commission's position that interruptions take precious time and can lead to important information not being transferred from one shift to the next. Although AHRQ (2013) did not mention the word interruption in their BSR implementation handbook, this study found that one way to minimize interruptions is to conduct BSR using a modified approach, where a portion of the hand‐off occurs inside and outside the patient's room. This need for a modified approach is further validated by other qualitative studies in the literature. Although the Joint Commission and AHRQ both provide broad descriptions of the critical content that should be included in hand‐off, this study identified the preferred location, inside or outside the patient's room, where specific critical topics should be discussed.

Results from this study have expanded the existing body of knowledge on BSR and should be used to inform its practice so the desired outcomes of patient and family satisfaction, nursing quality and patient safety, can be realized. This study should influence future research aimed at identifying strategies for successful implementation of BSR successfully. This study further validates the usefulness of the iPARIHS framework in exploring why innovations have not been implemented successfully.

## CONFLICT OF INTEREST

None.

## AUTHOR CONTRIBUTIONS

Joseph Jimmerson was responsible for the execution, scientific integrity, and administration of the study. He was responsible for the all correspondence with the designated study site, IRB, participants, and transcription services. He recruited participants, obtained consents, interviewed participants, sent recorded interviews to transcription service, and coded and analysed the data. He was ultimately responsible for monitoring the integrity of the data, study progress, and ensured the study protocol was followed. He prepared the findings for publication and is to be listed as first author. Patricia Wright was responsible for coding the first three interviews from both participant groups; independent from the primary investigator. She met with the PI to check for agreement in coding and to establish a codebook. The PI took that codebook, coded all remaining interviews, and established a final codebook with themes. She ensured that the themes identified correlated with the exemplar quotes provided and her understanding of the hand‐off process at UAMS. All discrepancies identified were investigated and a consensus between all investigators were reached before submission for publication. She aided the PI in preparing the findings for publication and is to be listed as second author. Patricia A. Cowan was responsible for reviewing and critiquing the codebook created by the Principle Investigator Joseph Jimmerson and Co‐investigator Patricia Wright. She ensured that the themes identified correlated with the exemplar quotes provided and her understanding of the hand‐off process at UAMS. All discrepancies identified were investigated and a consensus between all investigators were reached before submission for publication. She aided the PI in preparing the findings for publication and will be listed as third author. Tammy King‐Jones was responsible for reviewing and critiquing the codebook created by the Principle Investigator Joseph Jimmerson and Co‐investigator Patricia Wright. She ensured that the themes identified correlated with the exemplar quotes provided and her understanding of the hand‐off process at UAMS. All discrepancies identified were investigated and a consensus between all investigators were reached before submission for publication. She aided the PI in preparing the findings for publication and will be listed as fourth author. Claudia J. Beverly was responsible for reviewing and critiquing the codebook created by the Principle Investigator Joseph Jimmerson and Co‐investigator Patricia Wright. She ensured that the themes identified correlated with the exemplar quotes provided and her understanding of the hand‐off process at UAMS. All discrepancies identified were investigated and a consensus between all investigators were reached before submission for publication. She aided the PI in preparing the findings for publication and will be listed as fifth author. Geoffrey Curran was responsible for reviewing and critiquing the codebook created by the Principle Investigator Joseph Jimmerson and Co‐investigator Patricia Wright. He ensured that the themes identified correlated with the exemplar quotes provided and his understanding of the hand‐off process at UAMS. All discrepancies identified were investigated and a consensus between all investigators were reached before submission for publication. He aided the PI in preparing the findings for publication and will be listed as the six author.

## Supporting information

Appendix S1

## Data Availability

Additional information regarding this study, including transcribed interviews, are available and can be requested via the following email: jjimmerson@uams.edu.
